# Antioxidant and Anti-Inflammatory Properties of a *Thuja occidentalis* Mother Tincture for the Treatment of Ulcerative Colitis

**DOI:** 10.3390/antiox8090416

**Published:** 2019-09-19

**Authors:** Miruna Silvia Stan, Sorina Nicoleta Voicu, Sonia Caruntu, Ionela Cristina Nica, Neli-Kinga Olah, Ramona Burtescu, Cornel Balta, Marcel Rosu, Hildegard Herman, Anca Hermenean, Anca Dinischiotu

**Affiliations:** 1 Department of Biochemistry and Molecular Biology, University of Bucharest, 91–95 Spl. Independentei, 050095 Bucharest, Romania; miruna.stan@bio.unibuc.ro (M.S.S.); sori.petrache@yahoo.com (S.N.V.); cristina.nica@drd.unibuc.ro (I.C.N.); anca.dinischiotu@bio.unibuc.ro (A.D.); 2 Institute of Life Sciences, Vasile Goldis Western University of Arad, 86 Rebreanu, 310414 Arad, Romania; sonia.caruntu@gmail.com (S.C.); baltacornel@gmail.com (C.B.); ramrosu@gmail.com (M.R.); hildegard.i.herman@gmail.com (H.H.); 3 Faculty of Pharmacy, Vasile Goldis Western University of Arad, 86 Rebreanu, 310414 Arad, Romania; neliolah@yahoo.com; 4 SC PlantExtrakt SRL, 407059 Radaia, Cluj, Romania; ramona.burtescu@plantextrakt.ro; 5 Department of Histology, Faculty of Medicine, Vasile Goldis Western University of Arad, 86 Rebreanu, 310414 Arad, Romania

**Keywords:** *Thuja occidentalis*, ulcerative colitis, TNBS, antioxidants, inflammation, polyphenols

## Abstract

Inflammatory bowel disease (IBD) represents a group of chronic autoimmune and idiopathic disorders that are characteristic of industrialized countries. In contrast to drug therapies, which exert several side effects, herbal remedies have constantly attracted the attention of researchers. Therefore, in the present study, a mother tincture (MT) from fresh, young, non-woody *Thuja occidentalis* L. branches with leaves was obtained using distillation-based techniques. Further, this was used to assess its in vitro and in vivo antioxidant activities and anti-inflammatory properties, and to validate it as a potential phytotherapeutic treatment for IBD. The characterization of the tincture included common phytochemical screening assays for antioxidant capacity measurement, cell viability assays on Caco-2 colon cells, and in vivo assessment of antioxidant and anti-inflammatory effects by histopathological and ultrastructural analysis of the intestinal mucosa, measurement of reduced glutathione, lipid peroxidation, and gene expression of the inflammation markers (interleukin-6 and tumor necrosis factor-α) in intestine after oral administration to an experimental mouse model of colon inflammation (colitis) developed by intrarectal administration of 2,4,6-trinitrobenzenesulfonic acid (TNBS). Our study proved that administration of 25 or 50 mg *T. occidentalis* MT/kg of body weight/day by gavage for 7 days succeeded in inhibiting the inflammatory process induced by TNBS in the intestine, most probably because of its rich contents of flavonoids and phenolic compounds. These data could contribute to the formulation of therapeutic products based on *T. occidentalis* that could come to the aid of IBD patients.

## 1. Introduction

Ulcerative colitis (UC) is an inflammatory bowel disease that causes inflammation and ulcers in the lining of the colon and rectum. The symptoms include abdominal pain and diarrhea mixed with blood [1]. Although the etiological factors of this condition are not fully known, its occurrence is thought to be determined by genetic predisposition combined with environmental factors [2]. This disease is characteristic of industrialized countries, with its incidence being decreased in less developed countries. Systemic studies have shown that over 1.5 million people in North America are affected by inflammatory bowel disease (IBD), while over 2 million people are estimated to be affected in Europe [3]. Studies have also highlighted that high consumption of milk, sugar, food additives, animal proteins, and polyunsaturated fatty acids represent a risk factor in the development of IBD [4,5]. 

Although novel therapies are currently being developed and additional drugs have recently been approved for IBD treatment [6,7,8], there are several side effects that are closely related to the dose and the patient’s age [9]. For example, immunomodulators (azathioprine and 6-mercaptopurine) are the most effective treatment for IBD, but over time patients develop an increased risk of lymphoma [10,11]. Also, the use of infliximab—a tumor necrosis factor-α blocking agent—for the treatment of IBD in young patients is associated with hepatosplenic T cell lymphoma [12]. As IBD is initiated by oxidative stress that causes lesions in the mucosal layer of the gastrointestinal tract and promotes bacterial invasion that stimulates the immune response [13], herbal remedies rich in antioxidants represent an important source of new pharmacologically active compounds that have greater efficacy, lower toxicity, and less side effects. Natural products derived from *Aloe vera*, *Triticum aestivum*, *Boswellia serrata*, *Artemisia absinthium*, or *Tripterygium wilfordii* have been used since ancient times for the treatment of various inflammatory illness, including IBD, due to their high contents of antioxidants with anti-inflammatory actions [14]. 

Known as “thuja”, “white cedar”, or “tree of life” (arbor vitae), *Thuja occidentalis* L. is a coniferous tree originating in Canada and North America, which is grown as an ornamental tree in Europe, including Romania. In folk medicine, thuja has been used to treat diseases of the respiratory system (bronchial catarrh), urinary and reproductive systems (enuresis, cystitis, amenorrhea), as well as rheumatic and autoimmune diseases (psoriasis) [15]. Currently, thuja is used in homeopathy as a mother tincture (MT) or in diluted form [16].

The leaf branches of *T. occidentalis* L., being rich in tannins, flavonoids, polysaccharides, ethereal oil, and bitter principles, are the most valuable components of the plant in phytotherapy. However, bioactive compounds of the essential oils (diterpenes, as well as the monoterpenes α-thujone, β-thujone, fenchone, and sesquiterpene) characteristic of the *Thuja* genus are the main factor that gives the plant its many pharmacological and therapeutic effects, including antioxidant, antitumor, antibacterial, antifungal, antiviral, antiulcer, antipsychotic, emmenagogic, diuretic, expectorant, emollient, and hepatoprotective effects [17]. 

Thujone (C_10_H_16_O), the monoterpene with a ketonic structure, which is present in important amounts in *T. occidentalis*, is found in nature in the form of a mixture of two diastereoisomers, α-thujone and β-thujone. It represents the main compound of the essential oil extracted from the dry mass of the leaf branches, representing 60% of the compounds in the essential oil, which ranges between 1.4% and 4% in strength. The concentration of essential oil depends on the techniques used to isolate it. Thus, in the present study, a mother tincture of fresh, young, non-woody *T. occidentalis* L. branches with leaves was obtained using distillation-based techniques. Further, this was used to assess both its in vitro and in vivo antioxidant and anti-inflammatory properties and to validate it as a potential phytotherapeutic treatment for IBD. 

## 2. Materials and Methods

### 2.1. Obtaining and Characterization of MT

Mother tincture (MT) preparation: The *T. occidentalis* MT was prepared according to the German Homeopathic Pharmacopoeia (GHP) and European Pharmacopoeia (EP) from fresh, young, non-woody branches with leaves, using 90% vol. ethanol as the solvent. The vegetal material was harvested from PlantExtrakt ecological culture in Radaia, Cluj county, in September 2016. The extraction was performed in cold conditions by maceration as follows: one part of the minced vegetal material was mixed with 1.4 parts of solvent for 10 days, for 10–20 min/day. After 10 days of maceration, the extract was separated and filtered. 

Physicochemical characterization of MT: Prior to its use in antioxidant tests, the organoleptic properties, the relative density (Anton Paar DMA35 digital densimeter, Graz, Austria), the dry residue or residue at evaporation (Kern thermoscale), and the ethanol content (by distillation) were determined according to the methods from EP. For the identification of phenolic and coumarin compounds, thin layer chromatography (TLC) analysis was performed on silica gel plates, with a 254 nm indicator as the stationary phase and with a mixture of toluene and diisopropyl ether (80:20, vol./vol.) as the mobile phase. The migration was performed over 10 cm with the application of 10 μL of MT and 10 μL of standard mixture containing borneol 1 mg/mL and thujone 1 mg/mL in methanol. Further, the plate was dried in air and sprayed with phosphomolybdenic acid solution 20 wt% in ethanol, heated for 5–10 min at 100–105 °C, and then observed in visible light. 

The total flavonoid and phenolic acid contents were determined by UV-Vis spectral methods, according to the Romanian Pharmacopoeia (edition X for flavonoids and edition IX for phenolic acids), using a Cintra 101 UV-Vis spectrophotometer (GBC Scientific Equipment Ltd., Braeside, Australia). 

To determine the total flavonoids content, 5 mL of sodium acetate solution wt. 10% and 3 mL of aluminum chloride solution wt. 2.5% were added to 1 mL of MT, then the volume was brought to 25 mL with methanol. In addition, 1 mL of MT with 8 mL of water was diluted to 25 mL with methanol and used as blank, while different concentrations of rutoside were used as standards. After 30 min, the measurements were performed at 430 nm. 

To determine the total phenolic acid content, 0.5 mL of phosphotungstenic reagent was added to 0.1 mL of MT and the volume was brought to 25 mL with sodium carbonate solution wt. 15%. In addition, 0.1 mL of MT diluted to 25 mL with sodium carbonate solution wt. 15% was used as blank, while different concentrations of caffeic acid were used as standards. After 2 min, the measurements were performed at 715 nm. 

The content in thujone was also evaluated by gas chromatography-mass spectrometry (GC-MS). A Dani Master GC-MS system was used, along with a SH-Rxi-5ms column with dimensions of 30 cm × 0.25 mm × 0.25 mm and nitrogen as carrier gas, with a 10 mL/min flow rate and gradient temperature. The electrospray ionization mass spectrometry (EIS MS) detector identified the compounds with molecular weights from 50 to 600 daltons, and the ion source was operated at 200 °C. Afterwards, 5 mL of MT and 0.1 mL of standard with concentrations ranging from 20 to 80 nL/mL thujone were injected. The thujone was separated after 6.7 min. The thujone was identified with a match factor of 846 using the National Institute of Standards and Technology (NIST MS) 2.2 spectra database.

### 2.2. Assessment of the MT’s Antioxidant Capacity

The 2,2-diphenyl-1-picrylhydrazyl radical (DPPH) test: The antioxidant capacity of MT was determined using the 2,2-diphenyl-1-picrylhydrazyl radical (DPPH) according to the method of Burits et al. [18]. Different MT concentrations were mixed with 0.04% DPPH at a ratio of 1:100. After 30 min of incubation in dark at room temperature, the absorbance (A) of samples and blank (where the MT was substituted by an equal volume of 70% ethanol) was measured at 517 nm using a FlexStation 3 multi-mode microplate reader (Molecular Devices LLC, San Jose, CA, USA). Radical scavenging capacity (RSC; expressed as a percentage) was calculated using the following formula:

$$\mathrm{RSC}(\%)=\frac{A_{blank}-A_{sample}}{A_{blank}}\times 100$$

Oxygen radical absorbance capacity (ORAC) test: ORAC assay was performed according to the method of Davalos et al. [19]. A volume of 20 μL of MT or phosphate buffer (for blank) was incubated with 120 μL of 70 nM fluorescein for 15 min in darkness at 37 °C. The peroxyl radical was generated by adding 60 µL of 12 mM 2,2’-azobis (2-amidino-propane) dihydrochloride (AAPH), which was freshly prepared before each test. After 80 min of incubation in darkness at room temperature, the fluorescence intensity (FL) was recorded (excitation wavelength = 485 nm; emission wavelength = 520 nm) for 30 min at intervals of one minute using the FlexStation 3 multi-mode microplate reader (Molecular Devices LLC, San Jose, CA, USA). In parallel, a standard curve was prepared with Trolox (6-hydroxy-2,5,7,8-tetramethylcroman-2-carboxylic acid) at concentrations ranging 0–100 μM (0, 12.5, 25, 50, 75, and 100 μM). The results were calculated using the following formula. 

$$\mathrm{The}\;\mathrm{value}\;\mathrm{of}\;\mathrm{ORAC}=\left(FL_{probe}-FL_{blank}\right)/\left(FL_{Trolox}-FL_{blank}\right)/(\frac{Trolox\;molarity}{Sample\;molarity})$$

This ORAC value can also be expressed as µmol Trolox equivalents per gram of dried weight (d.w.).

Griess test: The nitric oxide radical scavenging assay was performed according to the method of Marcocci et al. [20]. Prior to the start of the experiment, a stock solution of 100 mM sodium nitroprusside in saline phosphate buffer (PBS) was prepared. A volume of 0.19 mL of different concentrations of MT was treated with 0.01 mL of sodium nitroprusside, then incubated at room temperature for 2 h. After 30 min and 2 h, 0.2 mL of the sample was recovered and homogenized with Griess reagent (1% sulfanilamide, 2% H_3_PO_4_, and 0.1% naphthylenediamine). Absorbance was read immediately at 546 nm using a FlexStation 3 multi-mode microplate reader (Molecular Devices LLC, San Jose, CA, USA). The absorbances were extrapolated on a standard curve using different concentrations of sodium nitroprusside between 0 and 50 μM as standard, with dilutions treated under the same conditions as samples. 

Polyphenol content: The total polyphenol content was measured by the Folin–Ciocalteu method. The polyphenols from the *T. occidentalis* MT reacted with the Folin–Ciocalteu reagent, forming a blue complex that was quantified by spectrophotometric measurement at 760 nm. Gallic acid (3,4,5-trihydroxybenzoic acid) was used as a standard antioxidant for the spectrophotometric determination of antioxidant activity. Absorbance of the radical solution was proportionally diminished with gallic acid concentration. For this experiment, a 1.25 mg/mL gallic acid solution was used for the standard curve and 5 dilutions (0.25, 0.50, 0.75, 1, and 1.25 mg/mL) were prepared from this solution. Further, 250 μL of 1/10 diluted Folin–Ciocalteu reagent was added to 50 μL of sample, homogenized, and incubated for 1 min at room temperature. Subsequently, 750 μL of Na_2_CO_3_ solution wt. 7.5% was added, the final volume was brought to 5 mL, and then incubated in the dark for 2 h at 25 °C. Finally, the optical density was measured at 760 nm using distilled water as blank. The total concentration of polyphenols was calculated by extrapolation in the gallic acid standard curve. The concentration of total phenolic compounds was expressed in milligrams of gallic acid equivalents (GAE) per gram of dried weight (d.w.).

### 2.3. In Vitro Antioxidant Capacity Assessment

Cell culture: In this study, the Caco-2 human colon cell line was purchased from American Type Cell Culture (ATCC, catalog no. CCL-2102). The cells were cultured in DMEM culture medium supplemented with 10% fetal bovine serum in a humidified 5% CO_2_ atmosphere at 37 °C.

Culture treatment protocol: When the cells reached 80% confluence, a 0.25% (w/v) Trypsin - 0.53 mM ethylenediamine tetraacetic acid (EDTA) solution (Sigma-Aldrich, St. Louis, MO, USA) was used to detach the cells. The enterocytes were counted and cultured at a density of 3 × 10^4^ cells/cm^2^ into 24-well plates (for viability assay and intracellular reactive oxygen species (ROS) measurement) or in 25 cm^2^ culture flasks (for glutathione content and lipid peroxidation analysis) and allowed to adhere overnight. Afterwards, Caco-2 cells were incubated with different concentrations (5, 25, 50, and 100 μg/mL) of MT for 12, 24, and 48 h (for MTT assay), whereas for oxidative stress evaluation, two doses (25 and 100 μg/mL) and two intervals of exposure (24 and 48 h) were selected. The oxidative stress was also tested, both in the presence and absence of the oxidizing agent (250 μM H_2_O_2_). For each experiment, untreated cells (untreated control) and cells treated with the oxidant (250 μM H_2_O_2_) were used. 

Cell viability: It was assessed using the 3-(4,5-dimethylthiazol-2-yl)-2,5-diphenyltetrazolium bromide (MTT) test [21]. This method consists of reducing the yellow MTT compound to purple formazan crystals by mitochondrial dehydrogenase-NAD(P)H (nicotinamide adenine dinucleotide phosphate reduced) dependent on the metabolically active cells. Formazan is then solubilized with 100% isopropanol and the concentration is determined spectrophotometrically at 595 nm. The culture medium was removed from each well and 0.5 mL of 1 mg/mL MTT was added. After 2 h of incubation at 37 °C, the MTT solution was removed and the formazan crystals from each well were solubilized with 0.5 mL of isopropanol. The absorbance of the samples was measured at 595 nm using a FlexStation 3 multi-mode microplate reader (Molecular Devices LLC, San Jose, CA, USA).

Preparation of cell lysates: Cells were collected from culture flasks, washed with PBS, and the cell lysates were obtained by sonication (30 s × 3 times) on ice with an ultrasonic processor (Hielscher UP50H, Teltow, Germany). The homogenate was centrifuged at 3000× *g* for 10 min at 4 °C and the total proteic extracts (EPT) represented by supernatants were collected for biochemical assays.

Determination of protein concentration by Bradford method: Protein concentrations of cell lysates were determined using the Bradford reagent (Sigma-Aldrich, St. Louis, MO, USA) and bovine serum albumin (BSA) as protein standard. Sample absorbance was determined at 595 nm using a FlexStation 3 multi-mode microplate reader (Molecular Devices LLC). 

Malondialdehyde (MDA) level measurement: A precise and often used method for monitoring lipid peroxidation uses thiobarbituric acid (TBA) as a reactive substance. MDA–TBA adducts formed following the reaction between the MDA present in the biological sample and the TBA at 37 °C can be measured by the fluorimetric method. Briefly, 200 μL of cell lysate was mixed with 700 μL of 0.1 M HCl and incubated at room temperature for 20 min. Afterwards, 900 μL of 0.025 M TBA was added and incubated again for 65 min at 37 °C, at which time TBA–MDA products were formed. Finally, the relative fluorescence units (RFU) were recorded using a Jasco FP-750 fluorimeter (excitation wavelength = 520 nm and emission wavelength = 549 nm) (FP-750 Spectrofluorometer, Jasco, Tokyo, Japan) and converted to nmols of MDA using a standard curve of 1,1,3,3-tetramethoxypropane, with concentrations ranging 0–0.5 mM. Also, the MDA level in each sample was expressed as nmol of MDA/mg protein. 

Glutathione (GSH) level measurement: Determination of reduced glutathione (GSH) concentration was performed with the Glutathione Assay Kit from Sigma. Therefore, cell lysates were deproteinized with 5% 5-sulfosalicylic acid (SSA) (in a ratio of 1:1) and centrifuged at 3000× *g* for 5 min at 4 °C to remove the precipitated proteins. Further, the samples were incubated with 5,5’-dithiobis-2-nitrobenzoic acid (DTNB) at room temperature for 5 min to allow the reduction of DTNB to 5-thio-2-nitrobenzoic acid (TNB) under GSH action. The optical density of the samples was measured at 412 nm using a microplate reader (TECAN GENios, Grödic, Germany). The GSH content was calculated by extrapolation on a GSH standard curve and expressed as nmols/mg protein.

### 2.4. In Vivo Antioxidant and Anti-Inflammatory Capacity Assessment

Experimental design: Animals used for this experiment were adult male CD1 mice weighing 25–30 g, raised in the animal facility of the Western University “Vasile Goldiş” in Arad. Food was withdrawn the day before the mice were sacrificed. Surgical interventions were adapted according to the International Ethical Guidelines for Animal Care. The project was authorized according to emergency order (OG) no. 42/2004 (the National Sanitary Veterinary and Food Safety Authority (ANSVSA) authorization no. 001/12.08.2016). The experimental animal model of intestinal inflammation (colitis) was performed by intrarectal administration of 1 mg of 2,4,6-trinitrobenzene sulfonic acid (TNBS) dissolved in 0.1 mL of 50% ethanol [22]. We selected three different doses of *T. occidentalis* MT (5, 25, and 50 mg MT/kg of body weight) based on our preliminary tests and in accordance with the previous published reports related to the in vivo anti-inflammatory and antioxidant potential of this tincture [23,24,25]. 

For this experiment, 100 animals were divided into 5 batches: *group 1* (control): on the first day 50% ethanol (~70–80 μL) was administered intrarectally, and for the next 7 days the group was administered 5% ethanol gavage solution/day (~300 μL); *group 2* (TNBS): on the first day 50 mg TNBS/kg of body weight was administered intrarectally, and for the next 7 days the group was administered 5% ethanol gavage solution/day; *group 3* (TNBS + 5 mg *T. occidentalis* MT/kg of body weight): on the first day 50 mg TNBS/kg of body weight was administered intrarectally, and for the next 7 days the group was administered gavage with 5 mg dry substance *T. occidentalis* MT/kg of body weight/day; *group 4* (TNBS + 25 mg *T. occidentalis* MT/kg of body weight): on the first day 50 mg TNBS/kg of body weight was administered intrarectally, and for the next 7 days the group was administered gavage with 25 mg dry substance *T. occidentalis* MT/kg body weight/day; *group 5* (TNBS + 50 mg *T. occidentalis* MT/kg of body weight): on the first day 50 mg TNBS/kg of body weight was administered intrarectally, and for the next 7 days the group was administered gavage with 50 mg dry substance *T. occidentalis* MT/kg of body weight/day. On the ninth day all mice were sacrificed, and tissue samples were taken and used for biochemical, histopathological, ultrastructural, and molecular biology analyzes. 

Histopathology analysis: Colon biopsies of all five experimental lots were fixed in 4% formalin in phosphate buffer, included in paraffin, and then cut to 4 μm using a Leica RM2125 RTS microtome (Leica Microsystems GmbH, Wetzlar, Germany). The histological sections were stained with hematoxylin and eosin (H&E) to analyze histopathological changes, or with Alcian blue to investigate the structural analysis of goblet cells. Analysis of the sections was performed on an Olympus BX43 optical microscope and captured using an Olympus XC30 digital camera and the Olympus Cell Dimension software.

Transmission electron microscopy (TEM) analysis: Colon biopsies were fixed in glutaraldehyde, washed in 0.1 M phosphate buffer, and post-fixed in 2% osmic acid in 0.15 M phosphate buffer. The dehydration was carried out in acetone and then the samples were included in Epon 812 resin. Ultra-sections of 60 nm thickness were made with a Leica EM UC7 ultramicrotome (Leica Microsystems GmbH, Wetzler, Germany) and then analyzed on an electron microscope with a Tecnai 12 Biotwin transmission (FEI company, Eindhoven, The Netherlands).

Real-time polymerase chain reaction (RT-PCR) analysis: Samples of intestinal tissue were stored in RNA solution (Thermo Scientific, Waltham, MA, USA) at −80 °C until analysis. The tissue was homogenized, and the RNA was extracted using the SV Total RNA Isolation System kit (Promega, Madison, WI, USA) according to the manufacturer’s instructions. The quantity and quality of extracted RNA were evaluated using the NanoDrop 8000 Spectrophotometer (Thermo Scientific, Waltham, MA, USA). Then, 2 μg total RNA was reverse transcribed into cDNA using the First Strand cDNA Synthesis Kit (Thermo Scientific, Waltham, MA, USA). The conditions for the reverse transcription reaction were: 25 °C for 5 min, 37 °C for 60 min, and 70 °C for 5 min. Real-time polymerase chain reaction (RT-PCR) was performed using the Maxima SYBR Green/ROX qPCR master mix kit (Thermo Scientific, Waltham, MA, USA) and the Mx3000P™ RT-PCR system (Thermo Scientific, Waltham, MA, USA). Each sample was analyzed in triplicate. The sequence of primers that was used and the validation data were obtained from the Primer Bank website (http://pga.mgh.harvard.edu/primerbank/) [26]. The amount of mRNA was normalized with glyceraldehyde-3-phosphate dehydrogenase (GAPDH). Relative changes in gene expression were determined using the 2-ΔΔCT method. The primers used were synthesized by Eurogentec (Liège, Belgium) and have the following sequences.


**Gene**

**Sense Sequence**

**Antisense Sequence**

**Tumor necrosis factor-alpha (TNF-ᾳ)**
5′CTGTAGCCCACGTCGTAGC3′5′TTGAGATCCATGCCGTTG3′
**Interleukin-6 (IL-6)**
5′AAAGAGTTGTGCAATGGCAATTCT 3′5′AAGTGCATCATCGTTGTTCATACA3′
**Glyceraldehyde-3-phosphate dehydrogenase (GAPDH)**
5′CGACTTCAACAGCAACTCCCACTCTTCC3′5′TGGGTGGTCCAGGGTTTCTTACTCCTT3′

Protein extraction: Protein extracts were obtained from the intestines of mice treated with TNBS and *T. occidentalis* MT. Briefly, 0.1 mg tissue was mixed with 1 mL of 0.1 M TRIS-HCl buffer and 5 mM EDTA (pH = 7.4), giving a 1:10 (m/V) homogenate. Further, the cell membranes were ruptured using the RETHSCH MM301 metallic ball homogenizer in two cycles of 2 min at 30 vibrations/sec, with 5 min rest on ice between the two mixes. The obtained homogenate was transferred to a new tube to remove the balls and centrifuged at 10,000 rpm/10 min/4 °C. The supernatant was transferred to new tubes and used immediately for biochemical determinations (GSH and MDA measurement). 

Determination of protein concentration by Lowry method: Protein concentration from intestinal tissues was determined by mixing 200 μL of each EPT, with an equal volume of alkaline copper reagent (prepared from 10 parts of reagent A and a reactive part B; where reagent A is a solution of 10% anhydrous Na_2_CO_3_ prepared in 0.5N NaOH, and reagent B is a 0.5% CuSO_4_·5H_2_O solution prepared in 1% sodium citrate solution). After 10 min of rest at room temperature, 600 μL of Folin-Ciocalteu reagent (diluted 10 times in distilled water) were added and incubated for 10 min at 50 °C. Finally, the optical density of the samples was measured at 660 nm using a Specord 200 Plus (Analytik Jena, Jena, Germany) spectrophotometer. The protein concentration of the samples was calculated by extrapolation in a BSA standard curve. 

MDA and GSH measurements were performed using the same methods as previously described in the “In vitro antioxidant capacity assessment“ section. 

### 2.5. Statistical Analysis

All data were expressed as mean values ± standard deviation (SD) of three different experiments. The statistical comparisons were performed by Student’s *t*-test or one-way analysis of variance (ANOVA), followed by Bonferroni‘s post-hoc test using Graph Pad Prism software (version 5; GraphPad Software, Inc., La Jolla, CA, USA). The results were considered statistically significant only if the *p*-values were less than 0.05. 

## 3. Results

### 3.1. Characterization of Thuja occidentalis MT

**Figure 1:**
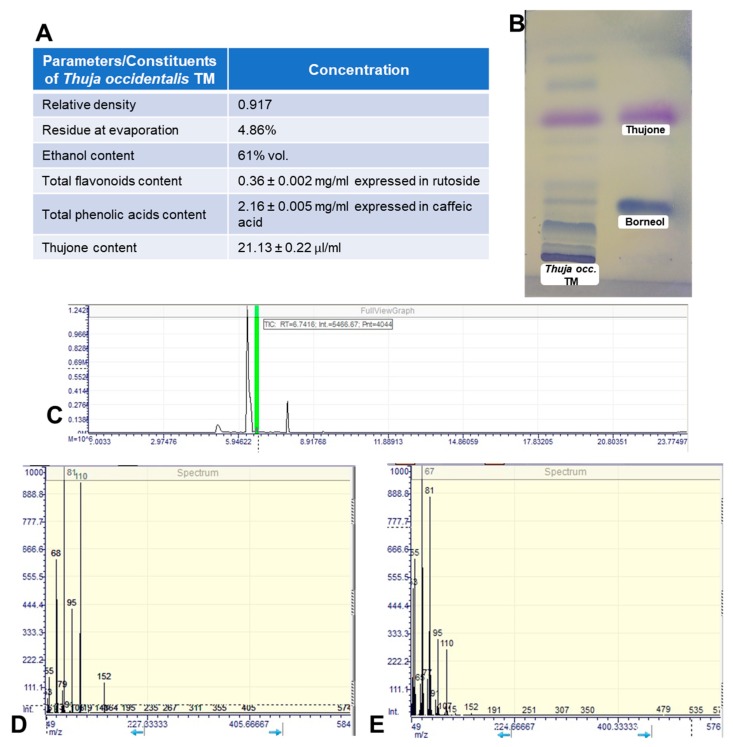
Characterization of the obtained *Thuja occidentalis* mother tincture (MT). (**A**) The main characteristics of *Thuja occidentalis* MT. (**B**) Thin layer chromatography (TLC) of *Thuja occidentalis* MT (left column) and standards (right column). (**C**) Gas chromatography (GC) chromatogram of *Thuja occidentalis* MT (green mark for thujone). (**D**) Mass spectrometry (MS) spectra of thujone from *Thuja occidentalis* MT. (**E**) MS spectra of standard thujone.

The main characteristics of the obtained MT with a greenish-brown color are shown in Figure 1A. The TLC chromatogram can be observed in Figure 1B. More blue or violet bands appeared because of the presence of terpenic compounds. In the upper third of the chromatogram, the violet bands of thujone can be clearly identified by comparison with the standards and MT chromatograms, while the weak blue band in the lower part indicates the presence of the borneol at a lower concentration. These two bioactive compounds are specific to thuja species. Previous in vitro and in vivo studies reported significant antioxidative and anti-inflammatory activities for thujone [27] and borneol [28,29], proving that these two compounds are responsible for the therapeutic effects of *T. occidentalis* MT.

### 3.2. Evaluation of the Antioxidant Capacity of Thuja occidentalis MT

The antioxidant potential of the tincture was estimated using four methods (Table 1): DPPH free radical scavenging activity, ORAC assay, NO radical scavenging test, and polyphenol content measurement. *T. occidentalis* MT exhibited 88.3% DPPH scavenging activity and almost 78% NO radical scavenging capacity, confirming that this tincture can react with DPPH and NO radicals, acting as free radical scavengers, as assessed by hydrogen donating ability [30]. In addition, the tincture was an effective scavenger of the 2,2’-azino-bis(3-ethylbenzothiazoline-6-sulphonic acid) (ABTS) radical, as measured by ORAC test (Table 1). The antioxidant protection could be conferred by the polyphenol content (3.9 mg GAE/g d.w.) of this tincture that can neutralize free radicals and inhibit the propagation of free radical reactions.

### 3.3. In Vitro Biocompatibility Screening

**Figure 2:**
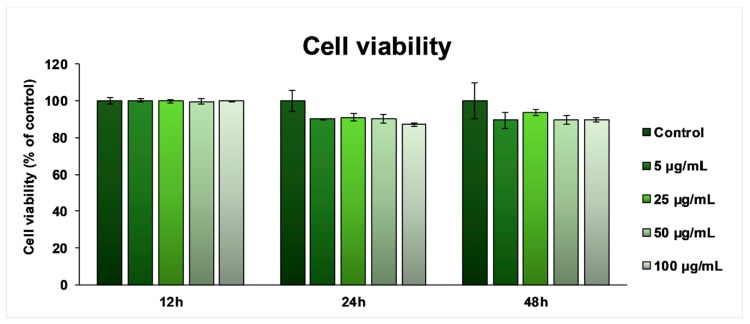
Biocompatibility of *Thuja occidentalis* MT as shown by cell viability assay after 12, 24, and 48 h incubation of the intestinal Caco-2 cells with different concentrations (5, 25, 50, and 100 μg/mL) of the selected tincture. Results are expressed as the mean ± standard deviation (SD) (*n* = 3) and represented relative to the untreated cells (control).

In order to assess cell viability, the Caco-2 cells were treated for 12, 24, and 48 h with different concentrations of *T. occidentalis* MT. After 12 h of exposure, the MTT test results (Figure 2) showed that cell viability remained unchanged compared to the untreated cells. After 24 h only, the viability of the cells incubated in the presence of the *T. occidentalis* MT showed a slight decrease of ~10% for all of the tested doses, which was maintained until 48 h, proving that the tincture had no harmful effects on the intestinal cells. 

**Figure 3:**
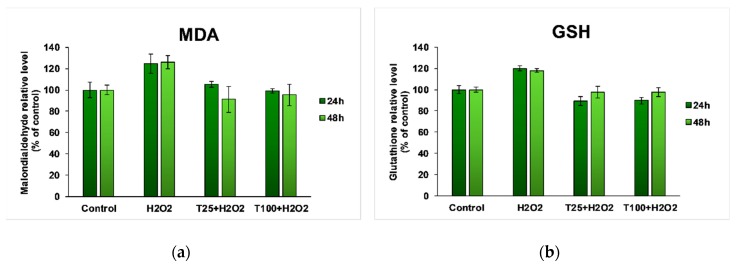
Malondialdehyde (MDA) (**a**) and reduced glutathione (GSH) (**b**) levels in Caco-2 cells exposed to different concentrations of *Thuja occidentalis* MT for 24 and 48 h in the presence of H_2_O_2_. Results are expressed as the mean ± standard deviation (SD) (*n* = 3) and represented relative to the untreated cells (control).

Oxidative stress was tested in the presence of an oxidizing agent (H_2_O_2_ 250 μM). For each experiment, untreated Caco-2 cells (control) and cells treated with H_2_O_2_ 250 μM were used. The presence of H_2_O_2_ in the culture media for both 24 and 48 h resulted in an increase of the MDA level by ~20% compared to untreated cells (Figure 3a). Following treatment with *T. occidentalis* MT, it was observed that the MDA concentration in the intestinal cells returned to the control value, and there were no significant differences between the 25 and 100 μg/mL doses. In contrast, after 48 h of treatment, there was a more visible decrease in the MDA level. 

Figure 3b shows an enhancement of GSH content in Caco-2 cells exposed to H_2_O_2_, proving the activation of the intrinsic GSH-based antioxidant mechanism for a proper cellular defense against oxidative damages. In the case of cells treated with *T. occidentalis* MT and H_2_O_2_ for both 24 and 48 h, the GSH level was maintained around the control value, proving its radical scavenging and antioxidant properties.

### 3.4. In Vivo Anti-Inflammatory Effect

#### 3.4.1. Histopathological Analysis of the Intestinal Mucosa

**Figure 4:**
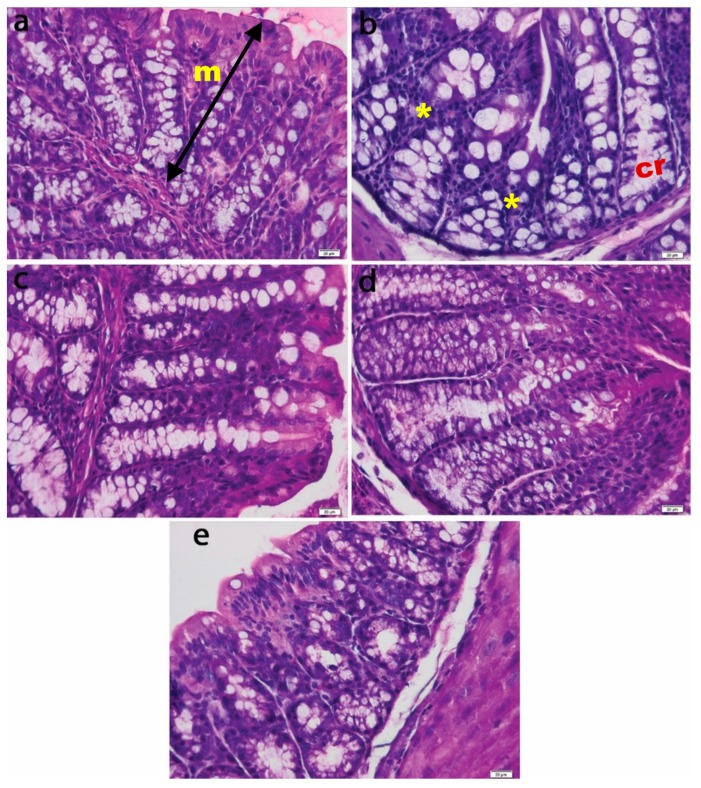
Histological appearance of intestinal mucosa in experimental groups: (**a**) Control; (**b**) 2,4,6-trinitrobenzenesulfonic acid (TNBS); (**c**) TNBS + 5 mg *Thuja occidentalis* MT/kg of body weight; (**d**) TNBS + 25 mg *Thuja occidentalis* MT/kg of body weight; (**e**) TNBS + 50 mg *Thuja occidentalis* MT/kg of body weight. Note: mucosa (m), crypt (cr), and inflammatory cells (*). Hematoxylin and eosin (H&E) staining 20×.

**Figure 5:**
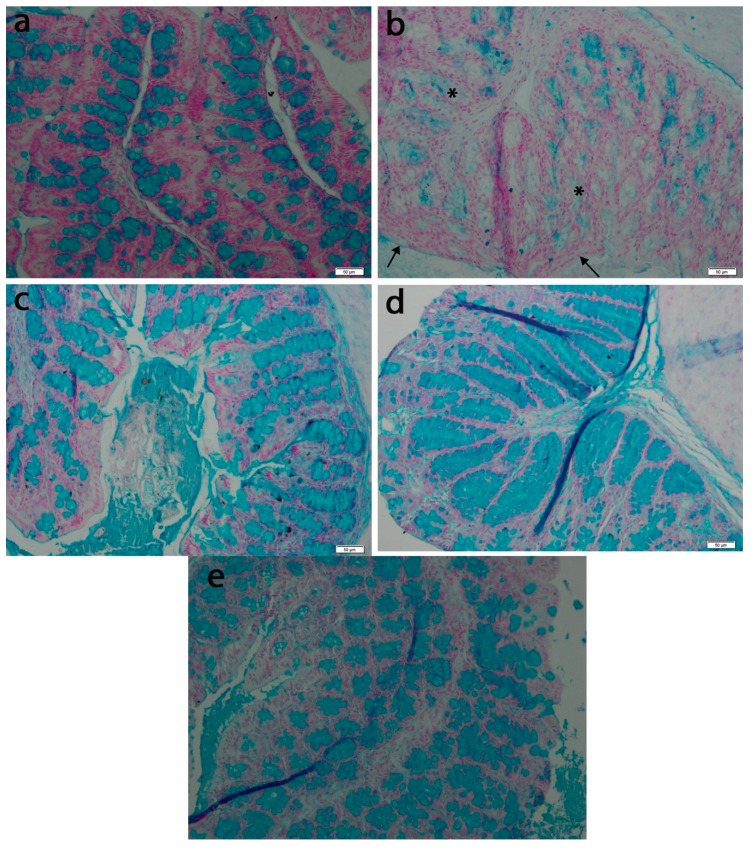
Appearance of goblet cells of intestinal mucosa in experimental groups: (**a**) Control; (**b**) TNBS; (**c**) TNBS + 5 mg *Thuja occidentalis* MT/kg of body weight; (**d**) TNBS + 25 mg *Thuja occidentalis* MT/kg of body weight; (**e**) TNBS + 50 mg *Thuja occidentalis* MT/kg of body weight. Note: discontinuous layer of mucus at the luminal face of the epithelium (arrows), and goblet cells with a more pale and inconsistent mucus (asterisks). Alcian blue staining 50×.

H&E colonic mucosa analysis of the non-colitic group revealed straight tubular glands and normal aspects of epithelial and goblet cells. In the TNBS control group, tubular glands were reduced and the presence of inflammatory infiltrate was observed (Figure 4). The animals who were given oral *T. occidentalis* MT showed a progressive restoration of the colonic mucosal histoarchitecture, with a decrease in inflammatory infiltrate, straight glands, and normal crypt, showing almost similar values to those of healthy animals (Figure 5). 

Histological analysis of the control group using Alcian blue staining revealed the presence of normal looking mucosa with straight tubular glands, which contained mucous cells with acidic and neutral mucins, filling a continuous layer of mucus at the luminal face of the epithelium (Figure 5a). The crypts and luminal epithelium had a normal appearance. In contrast, in the TNBS group, the goblet cells presented a more pale and inconsistent mucus content, with the mucus layer becoming thinner and discontinuous (Figure 5b). These morphological changes—typical for colon inflammation were attenuated by treatment with *T. occidentalis* MT—and the protective effects increased with the administered dose.

#### 3.4.2. Analysis of Specific Inflammation Markers of the Intestinal Mucosa

**Figure 6:**
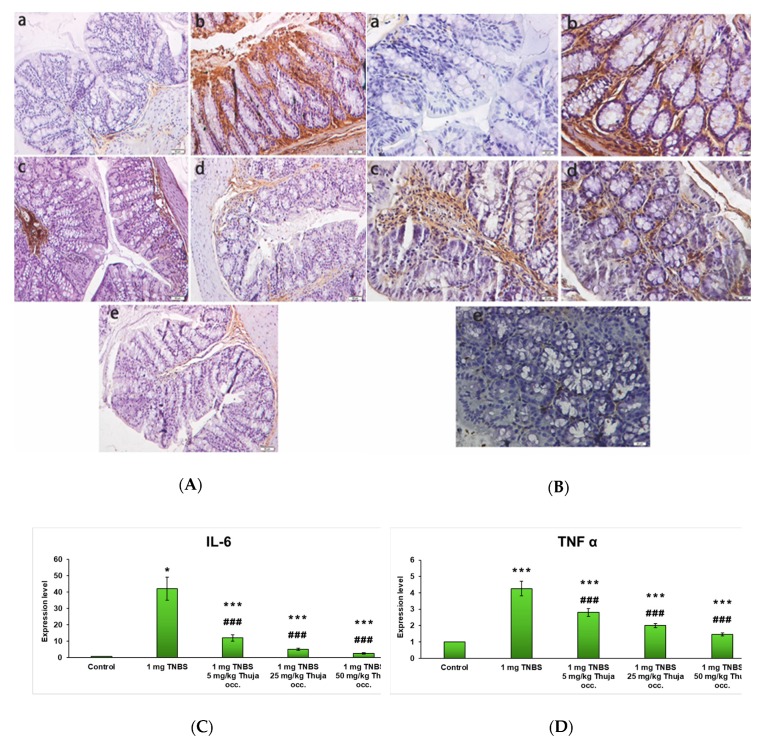
Immunohistochemical expression of IL-6 (**A**) and TNFα (**B**) in the intestinal mucosa for experimental groups: (**a**) Control; (**b**) TNBS; (**c**) TNBS + 5 mg *Thuja occidentalis* MT/kg of body weight; (**d**) TNBS + 25 mg *Thuja occidentalis* MT/kg of body weight; (**e**) TNBS + 50 mg *Thuja occidentalis* MT/kg of body weight. Magnification at 20× (**a**) and 50× (**b**,**c**). Gene expression of interleukin-6 (IL-6) (**C**) and tumor necrosis factor-alpha (TNF-α) (**D**) inflammation markers in the experimental groups. Note: *** *p* < 0.001 versus control; ^###^ *p* < 0.001 versus TNBS group.

Intrarectal administration of the toxic substance (TNBS) led to a significant increase in the immunohistochemical expression of the inflammation markers interleukin-6 (IL-6) (Figure 6A) and tumor necrosis factor-alpha (TNF-α) (Figure 6B) compared to control. Following the oral administration of the three doses of *T. occidentalis* MT (5, 25, and 50 mg/kg of body weight), the expression of the inflammation markers decreased compared to the mice intoxicated with TNBS, even becoming similar to the control for the 50 mg tincture/kg of body weight dose. 

The gene expression of IL-6 (Figure 6C) and TNF-α (Figure 6D) inflammation markers was significantly increased in the rectal toxicant (TNBS) group compared to the control (*p* < 0.001). Following the oral administration of 3 increasing doses of *T. occidentalis* MT (5, 25, and 50 mg tincture/ kg of body weight), the expression of the two markers of inflammation in the intestinal mucosa decreased significantly compared to the TNBS group (*p* < 0.001).

#### 3.4.3. Ultrastructural Analysis of the Intestinal Mucosa by TEM

**Figure 7:**
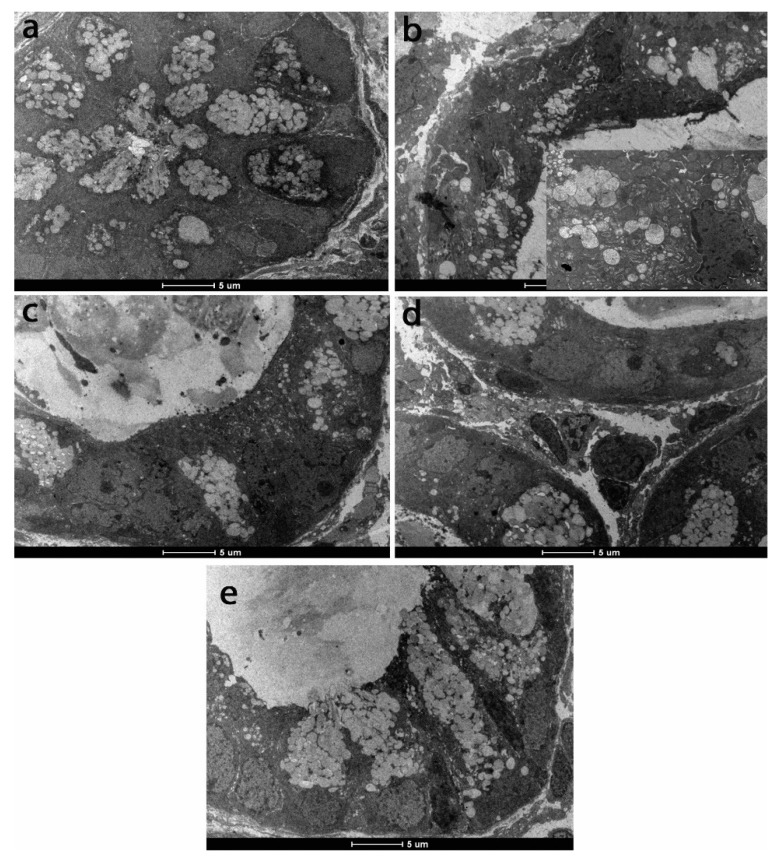
Ultrastructural appearance of intestinal mucosa in experimental groups: (**a**) Control; (**b**) TNBS; (**c**) TNBS + 5 mg *Thuja occidentalis* MT/kg of body weight; (**d**) TNBS + 25 mg *Thuja occidentalis* MT/kg of body weight; (**e**) TNBS + 50 mg *Thuja occidentalis* MT/kg of body weight.

The ultrastructural analysis of the TNBS group revealed the presence of altered epithelial and mucosal cells, the presence of apoptotic nuclei, the rarefied mitochondria matrix and altered cristae, the endoplasmic reticulum with dilated cisterns, and even lysis areas. A significant decrease in mucosal secretion for goblet cells was also noted (Figure 7). Ultrastructural alterations shown by TEM were gradually attenuated by increasing the dose of *T. occidentalis* MT, and even mucosal secretion was restored to the control level for the 25 and 50 mg/kg of body weight doses (Figure 7).

#### 3.4.4. Lipid Peroxidation and Antioxidant Defense in the Intestinal Mucosa

**Figure 8:**
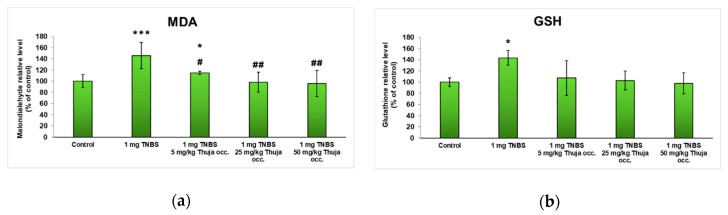
The level of malondialdehyde (MDA) (**a**) and reduced glutathione (GSH) (**b**) at the intestinal level in the experimental groups. Note: * *p* < 0.05 and *** *p* < 0.001 versus control; ^#^ *p* < 0.05 and ^##^ *p* < 0.01 versus TNBS group.

The degree of lipid peroxidation (Figure 8a) reaches significantly increased values for the experimental group with colitis induced by TNBS administration (a 60% increase over control), but oral treatment with *T. occidentalis* MT managed to reduce the lipid peroxidation, as evidenced by low values compared to the TNBS-treated group. The beneficial effect was best observed after administration of 25 or 50 mg of dried *T. occidentalis* MT/kg of body weight, bringing the MDA value almost to the control level (110% and 102%, respectively).

Regarding the level of the main intracellular antioxidant, GSH (Figure 8b), an increase of 44% was recorded after the induction of colitis with TNBS. However, after constant administration of 25 and 50 mg *T. occidentalis* MT/kg of body weight for one week, the GSH level was much lower compared to the TNBS group, reaching 103% and 98% of the untreated mice values (control), respectively. 

## 4. Discussion

For the present study, an experimental model of colon inflammation (colitis) was developed by intrarectal administration of 2,4,6-trinitrobenzenesulfonic acid (TNBS) in CD1 mice. TNBS was administered intrarectally to animal models and induced an immune response mediated by Th1 cells in the intestinal mucosa [31]. Thus, it resulted in inflammation of the mucosa characterized by infiltration of macrophages and T cells along the wall of the large intestine, as is shown in Figure 4b. Also, the crypts were reduced, the integrity of the membrane was lost, and the mucous cells had an atypical appearance with extensive edema (Figure 5b). TNBS-induced colitis included the secretion of various pro-inflammatory cytokines, such as IL-12, which played an important role in differentiation of T lymphocytes into Th1 effector cells that synthesize interferon gamma (IFN-γ) and TNF-α [32]. IFN-γ acted on macrophages, stimulating the synthesis of inflammatory mediators IL-6, IL-1β, and TNF-α, as confirmed in Figure 6B-b,C-b. After induction of colitis, animal models developed symptoms characteristic of this inflammatory bowel disease, such as weight loss, diarrhea, and bloody stools [33]. The onset and severity of symptoms varied depending on the species of animals used as experimental models and the dose of TNBS administered. However, the changes revealed within our study are in good agreement with the description in the review of Antoniou et al. [33]. 

Since its first description in 1989 by Morris et al. [34], the model of TNBS-induced colitis has been used for its similar properties to Crohn’s disease, an incurable and lifelong disease that deserves a more efficient treatment. New alternative approaches to current treatments have attracted the interest of scientists aiming to diminish the side effects associated with them. Research studies on this topic include the assessment of various treatments, such as hemin (a heme-oxygenase inducer) [35], vitamin D [36], lactate [37], anti-TNF monoclonal antibody [38] or plant-derived compounds (resveratrol [39], gallic acid [40], red seaweed *Porphyra vietnamensis* [41], or a 6-herb Chinese medicinal formula [42]), which have proven anti-inflammatory properties able to prevent or to ameliorate the severity of colitis. 

In this continuous search for new complementary and alternative formulations, we aimed to provide a complete evaluation of the antioxidant and anti-inflammatory effects of a mother tincture of *T. occidentalis*, an endemic and ornamental tree that has proved its therapeutic potential over the years [15]. Therefore, in the present work we were able to show that ORAC, DPPH, and NO scavenging activities (Table 1) obtained for this tincture could indicate *T. occidentalis* TM as a very good candidate in the search for natural, effective substances with antioxidant activity. In addition, our results are in agreement with previous studies on *T. occidentalis* extracts [30,43]. 

The anti-inflammatory effects exerted by *T. occidentalis* MT were investigated by histopathological and ultrastructural analyses of the intestinal mucosa, the immunohistochemical analysis of the inflammation markers (TNF-α, COX-2, and IL-6), and of lipid peroxidation and reduced glutathione in the intestine. The experiment carried out on the 5 groups of mice (control, 1 mg TNBS/kg of body weight, 1 mg TNBS + 5 mg *T. occidentalis* MT/kg of body weight, 1 mg TNBS + 25 mg *T. occidentalis* MT/kg of body weight, and 1 mg TNBS + 50 mg *T. occidentalis* MT/kg of body weight) showed that medium and high doses of tinctures managed to relieve intestinal inflammation experimentally induced by TNBS. The results obtained from the tests revealed that the tincture contributed to the alleviation of oxidative stress and attenuation of lipid peroxidation, a process activated in the intestinal inflammation chemically induced with TNBS. Restoration of the colonic mucosal histoarchitecture was demonstrated by a decrease in inflammatory infiltrate and an increase in mucosal secretion compared to TNBS changes. Ultrastructural alterations were progressively attenuated by increasing the dosage of the tincture, which was able to significantly decrease the production of IL-6 and TNF-α. Therefore, the protective effect of the mother tincture against colitis could be related to its ability to re-establish the level of inflammatory cytokines to near that of healthy control.

In parallel, the antioxidant and anti-inflammatory effects of *T. occidentalis* MT were tested in vitro on human Caco-2 colon cells. No toxic effect was induced on the intestinal cells, and after the exposure to H_2_O_2_, an inducer of oxidative stress and inflammation, the tincture proved its radical-scavenging and antioxidant properties, as the levels of MDA and GSH decreased to near control values. There is a good correlation between the composition of the mother tincture and the antioxidant and anti-inflammatory activities, with the phenolic and flavonoids compounds (Figure 1) being responsible for the attenuation of oxidative stress and decrease of inflammation in colon cells. Based on the TLC chromatogram represented in Figure 1B, we considered that thujone was one of the major active compounds found in *T. occidentalis* MT (as was also shown by a previous report [44]), being responsible for the antioxidant effect evidenced in the in vitro study. Thujone has the ability to inhibit lipid peroxidation [44] and to decrease nitric oxide and nuclear factor kappa B (NF-κB) production via macrophages [27]. In addition, the high content in borneol could explain the good results obtained for MDA and GSH, and also for pro-inflammation markers. Previously, it was reported that borneol was able to suppress the pro-inflammatory cytokine mRNA expression in colonic inflammation in mice [45]. Also, this bicyclic organic monoterpene reduced lipid peroxidation and increased enzymatic activities and non-enzymatic antioxidant levels in hypersensitive rats [46].

Our study provides new knowledge regarding the beneficial therapeutic properties of *T. occidentalis* TM for the treatment of ulcerative colitis through the in vitro and in vivo assessments performed on experimental inflammation models. Despite the data evidenced within our research, clinical confirmations are still needed for proper and safe administration in humans without any toxicological risks.

## 5. Conclusions

The environmental factors associated with the genetic predisposition of the individual can lead to the appearance of chronic autoimmune diseases, among them the inflammatory bowel diseases. Intestinal inflammation is the common symptom of these diseases and is associated with increased production of reactive oxygen and nitrogen species, and induction of oxidative stress. The therapeutic properties of *T. occidentalis* MT were best noted in orally administered medium and high doses, which succeeded in inhibiting the inflammatory process induced by TNBS in the intestine, most probably based on its rich contents of flavonoids and phenolic compounds. These data can contribute to the formulation of therapeutic products based on *T. occidentalis* that could patients who have an inflammatory bowel disease.

## Figures and Tables

**Table 1:** Evaluation of the antioxidant capacity of *Thuja occidentalis* MT.

Parameter	DPPH (% Inhibition)	ORAC (μmol Trolox/g d.w.)	NO (% Inhibition)	Polyphenols Content (mg GAE/g d.w.)
*Thuja occidentalis* MT	88.3 ± 1.54	50.8 ± 1.46	77.9 ± 3.45	3.9 ± 0.09

Values are expressed as means ± standard deviation (SD) (*n* = 3). Abbreviations: MT = mother tincture; DPPH = 2,2-diphenyl-1-picrylhydrazyl radical; ORAC = oxygen radical absorbance capacity; GAE = gallic acid equivalents.
